# Mortality and associated factors among children admitted to an intensive care unit in muhimbili national hospital, from the time of admission to three months after discharge: a prospective cohort study

**DOI:** 10.1186/s12887-024-04620-6

**Published:** 2024-03-08

**Authors:** Rehema E. Lyimo, Yasser H. Said, Sokoine L. Kivuyo, Deogratias Nkya, Francis F. Furia

**Affiliations:** 1 Department of Pediatrics and Child Health, School of Medicine, Muhimbili University of Health and Allied Sciences, Dar es Salaam, Tanzania; 2 Department of Paediatrics and Child Health, Muhimbili National Hospital, Dar es Salaam, Tanzania; 3 National Institute for Medical Research, Dar es salaam, Tanzania; 4 Department of Pediatric Cardiology, Jakaya Kikwete Cardiac Institute, Dar es salaam, Tanzania

**Keywords:** Mortality, Pediatric Intensive care unit and post-pediatric intensive care unit discharge

## Abstract

**Background:**

Mortality of children admitted to Intensive Care Units (ICU) is higher in low-to-middle-income countries (LMICs) as compared to high-income countries (HICs). There is paucity of information on outcomes following discharge from ICU, especially from sub-Saharan Africa region. This study was conducted to determine mortality and its associated factors among children admitted to Pediatric ICU (PICU) at Muhimbili National Hospital, from admission to three months after discharge.

**Methodology:**

This was a hospital-based prospective cohort study conducted between July 2021 and May 2022, among children admitted to PICU who were followed up for 3-month after discharge. Structured questionnaires were used to collect data from their medical charts. Telephone interviews were made after discharge. Medical records and verbal autopsy were used to determine the cause of death after discharge. Cox regression analysis was performed to assess the association between variables. A p-value of < 0.05 was considered statistically significant. Survival after PICU discharge was estimated by Kaplan - Meier curve.

**Results:**

Of 323 children recruited, 177(54.8%) were male, with a median age of 17 months (1-168). The leading cause of PICU admission was severe sepsis 90/323(27.9%). A total of 161/323 children died, yielding an overall mortality of 49.8%. Of 173 children discharged from PICU, 33(19.1%) died. The leading cause of death among children who died in the general ward or as readmission into PICU was sepsis 4/17(23.5%). Respiratory diseases 4/16(25.0%) were the commonest cause of death among those who died after hospital discharge. Independent predictors of overall mortality included single organ dysfunction with hazard ratio(HR):5.97, 95% confidence interval (CI)(3.05–12.26)] and multiple organ dysfunction [HR:2.77,95%CI(1.03–2.21)]. Chronic illness[HR:8.13,95%CI(2.45–27.02)], thrombocytosis [HR:3.39,95%CI(1.32–8.73)], single[HR:3.57,95%CI(1.42–9.03)] and multiple organ dysfunction[HR:3.11,95%CI(1.01–9.61)] independently predicted post-PICU discharge mortality.

**Conclusion:**

Overall mortality and post- PICU discharge mortality were high and more likely to affect children with organ dysfunction, chronic illness, and thrombocytosis. The leading causes of mortality post- PICU discharge were sepsis and respiratory diseases. There is a need for a focused follow up plan of children post- PICU discharge, further research on the long term survival and strategies to improve it.

## Background

Approximately 1.5% of hospitalized children require intensive care unit (ICU) [1], and among the leading causes of admission into ICU for children are infections and trauma [2]. Low-to-middle income countries have limited facilities for intensive care services for children, and they are also reported to have higher ICU mortality as compared to high-income countries [3]. Mortality among critically ill children is also reported to be high following ICU discharge [4–6].

Mortality following Pediatric Intensive Care Unit (PICU) discharge has been noted to be between 9.7% and 14% from studies conducted in high-income countries [4–6]. Currently, there are no published data in Sub- Saharan Africa that address the mortality of critically ill children after discharge from ICU but there are few studies which reported on mortality after hospital discharge [7–10].

Given this paucity of information in sub-Saharan Africa, this study was conducted among children admitted in PICU at Muhimbili National Hospital (MNH) in Dar es Salaam, Tanzania to determine mortality of children from the time of admission to three months after PICU discharge and its associated factors.

The results from this study will contribute valuable insights into the survival of children following critical illness. Additionally, these findings will contribute to the formulation of a follow-up protocol for children after critical illness in our specific setting. Moreover, they will serve as a reference for future studies in the field of pediatric intensive care units (PICU).

## Methodology

### Study design, duration, setting and participants

This was a hospital-based prospective cohort study conducted from July 2021 to May 2022 at pediatric intensive care unit (PICU) in Muhimbili National Hospital (MNH). MNH functions as a tertiary and university teaching hospital in Tanzania, catering to patients referred from various public and private centers. These individuals are admitted to the hospital’s pediatric surgical and medical general wards, which collectively offer a total of 298 beds.

PICU attends to children aged 1 month to 14 years who are admitted from the emergency department, as well as surgical and medical general wards.It is equipped with emergency medications, 12 ventilating machines, 5 specialists, 12 beds, and a maximum of 1:2 nurse-to-patient ratio. All patients discharged from PICU are transferred to general wards.

All children admitted in PICU and whose parents consented were conveniently recruited to a sample size of 323. Exclusions during the study period encompassed children who were readmitted, as they had already been included in their initial admission. Additionally, children who passed away within 24 h of admission were excluded due to incomplete investigations and information.

### Variables

#### Outcome variable

Mortality was the outcome of interest.

#### Independent variable

Age, admitting diagnosis, type of admission, chronic disease, laboratory parameters and organ dysfunction were variables which influenced the outcome.

#### Data collection method

Structured questionnaires were used for data collection which included social demographic characteristics, type of admission, admitting diagnosis, laboratory results, chronic illness, and organ dysfunction. Social demographic characteristics and admitting diagnosis were obtained from the hospital records within 24 h of admission. History taking, physical examination and laboratory tests were done within 24 h of admission.

Information on the presence of chronic illness was gathered through a research-administered interview, were chronic illness was defined as a condition lasting for three months or more. Organ dysfunction was assessed at admission and defined based on criteria of organ dysfunction as adapted and modified from Goldstein and Proulx were multiple organ dysfunction was defined as involvement of more than one organ dysfunction [11, 12]. Children admitted to PICU were followed up until death or discharge.

Those who were discharged from PICU were observed closely while in the general ward. Further, follow-up after hospital discharge was done at 3 months after PICU discharge through a telephone interview. Lost to follow-up was minimized by obtaining three phone numbers from parents or guardians. Participants were considered lost to follow-up after a lack of response from the caregivers for two consecutive weeks.

Post-PICU mortality included children who died in a general ward, as re-admission to PICU, or after discharge from the hospital. Details of post-PICU mortality which occurred prior hospital discharge at MNH was obtained from burial permits and the patient’s medical record.

Details of deaths that occurred after hospital discharge were obtained through telephone interview, using verbal autopsy which was adapted from WHO [13] and reviewed by two pediatricians before data collection.

#### Definition of organ dysfunction

##### Brain dysfunction

was GCS of < 11 or presence of fixed and dilated pupils.

##### Lung dysfunction

was oxygen saturation of less than 90% in patient without cardiac disease and below 75% for patients with cardiac disease which will be measured by pulse oximeter or need for mechanical ventilation or respiratory rate > 90 bpm for infants and > 70 for children above 1 year.

##### Cardiovascular system

was defined by either need of inotropes or presence of cardiac arrest or heart rate of < 50 or > 220 bpm for infants and < 40 or > 200 bpm for children above 1 year or systolic pressure of < 40 mmhg for infants and < 50mmhg for children above 1 year.

##### Liver dysfunction

was defined by the presence of jaundice and/or elevation of ALT twice or more above normal value.

##### Kidney dysfunction

was defined as elevation of creatinine above normal range for age or need for dialysis.

##### GIT dysfunction

was defined by severe GIT bleeding which need transfusion of blood products.

##### Hematological dysfunction

was defined by either HB < 5 g/dl or leucopenia of < 3000 cells/mm3 or thrombocytopenia of < 20,000cells/mm3.

#### Definition of other terms

##### Leucocytosis

Increase of white blood cells of > 11 × 10^9^/L.

##### Thrombocytosis

Increase of platelets of > 450 × 10^9^/L.

#### Data analysis

Data entry and analysis were done using SPSS version 25. Descriptive statistics were summarized in a frequency distribution table, pie chart, bar chart, median and interquartile range. Kaplan-Meier curves showed the survival of participants after PICU discharge. Association between variables was estimated using Cox regression analysis. After univariate analysis, factors with a p-value less than 0.2 were included in correlation matrix. Factors that had a correlation coefficient greater than 0.5 were considered to have a strong correlation. In such cases, only one of the two factors was included in the multivariate analysis. Crude and adjusted hazard ratio with 95% confidence interval were calculated and findings with p values < 0.05 after analysis were considered statistically significant.

## Results

**Fig. 1:**
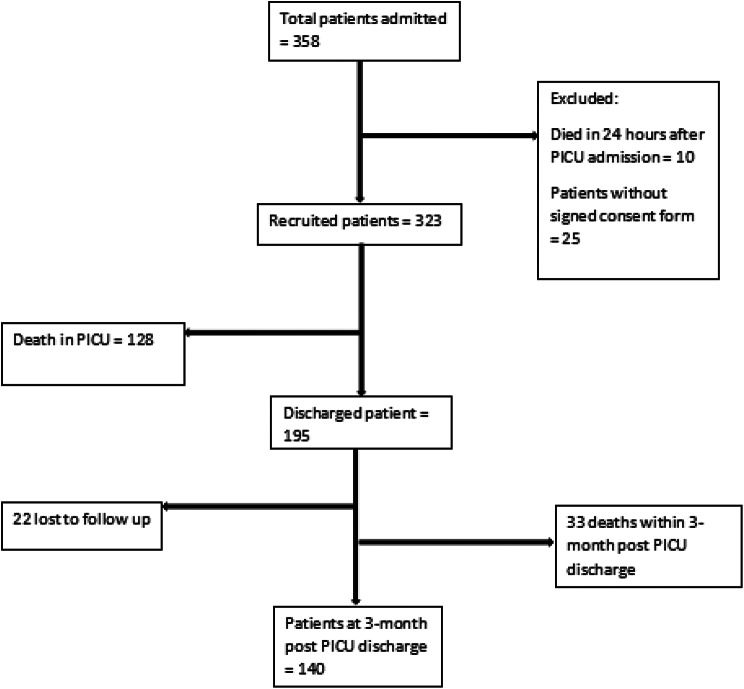
Flow chart on enrollment of study participants

Of 358 children admitted in pediatric intensive care unit, 323 were recruited and 35 were excluded due to different reasons as shown in Fig. 1.

### Socio-demographic characteristics of study participants

Three hundred and twenty-three participants were recruited, 177(54.8%) Males and 136(42.1%) aged between 1 and 5 years with a median age of 17 months and inter quarterly range (IQR) of 1–168months. Majority 238(73.7%) had a medical diagnosis with the commonest admitting diagnosis being severe sepsis 90(27.9%). Multiple organ dysfunction, and chronic illness were observed in 134(41.5%) and 139(43.0%) of participants respectively. Elevated creatinine and ALT levels were noted in 85(26.3%) and 61(18.9%) participants respectively. Hematological derangement noted among participants included anemia 235(72.8%), leukocytosis 181(56%), and thrombocytosis 43(13.3%). Table 1.

**Table 1:** Socio-demographic and clinical characteristics of study participants

Variable	Frequency (*n*)	Percent (%)
Age group (years)		
<1	120	37.2
1–5	136	42.1
>5	67	20.7
Sex		
Female	146	45.2
Male	177	54.8
Diagnosis		
S. Sepsis	90	27.9
S. Pneumonia	41	12.7
Meningitis	17	5.3
CKD	10	3.1
Malaria	16	5.0
AWD	15	4.6
Post-surgery	62	19.2
Burn injury	16	5.0
Others	27	8.4
PAIDS	12	3.7
DKA	9	2.8
Malignancy	8	2.5
Type of admission		
Medical	238	73.7
Surgical	85	26.3
Chronic illness		
Yes No	139	43.0
	184	57.0
Organ dysfunction		
Single	103	31.9
Multiple	134	41.5
None	86	26.6
Creatinine (micromole/L)		
Elevated	85	26.3
Normal	238	73.7
ALT (units/L)		
>114	61	18.9
<114	262	81.1
WBC (×10^9^/L)		
<4	8	2.5
>11	181	56.0
4–11	134	41.5
Hemoglobin (g/dl)		
<11	235	72.8
>11	88	27.2
Platelets (×10^9^/L)		
<150	90	27.9
>450	43	13.3
150–450	190	58.8

**S. Sepsis**: Severe sepsis, **S. Pneumonia**: Severe pneumonia, **CKD**: Chronic kidney disease, **AWD**: Acute watery diarrhea, **PAIDS**: Pediatric acquired immunodeficiency syndrome, **DKA**: Diabetic ketoacidosis, **ALT**: Alanine aminotransferase, **WBC**: White blood cells.

**NB**: Chronic illness: PAIDS, CKD, sickle cell anemia (SCA), malignancy, syndromic babies, diabetes, cerebral palsy (CP), congenital malformations, epilepsy, nephrotic syndrome and cystic hygroma

### Mortality rate from PICU admission to three months after discharge

**Fig. 2:**
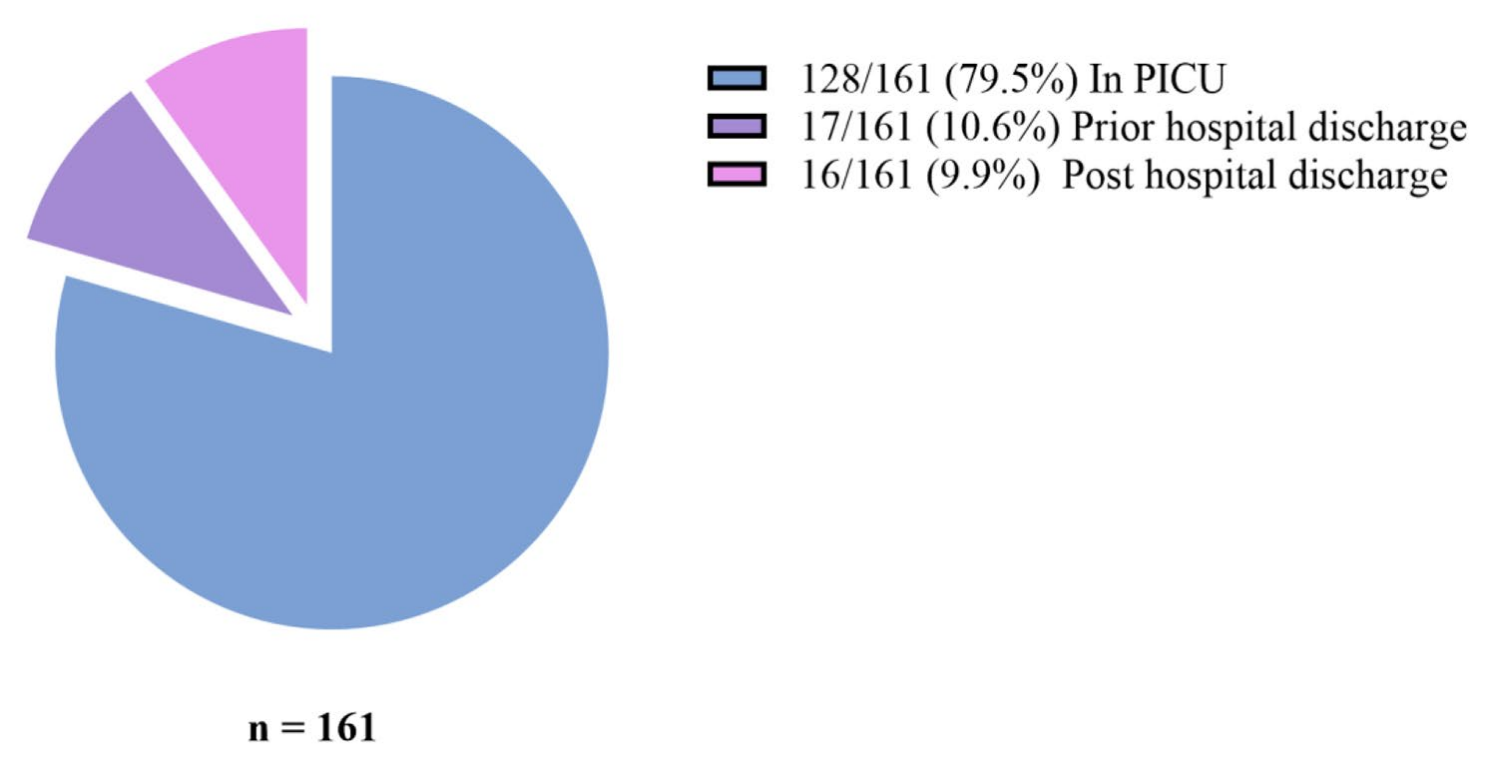
A Pie chart showing the place of death for children admitted to PICU

**Fig. 3:**
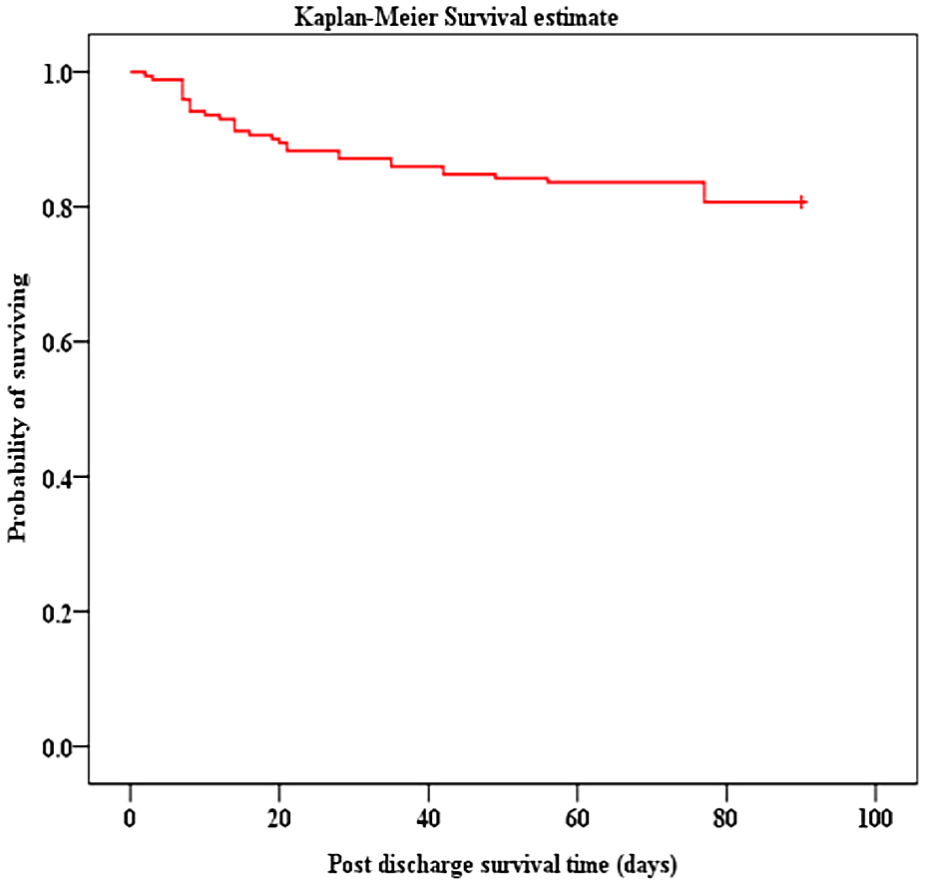
Kaplan-Meier Survival Curve for children who were discharged from PICU

**Fig. 4:**
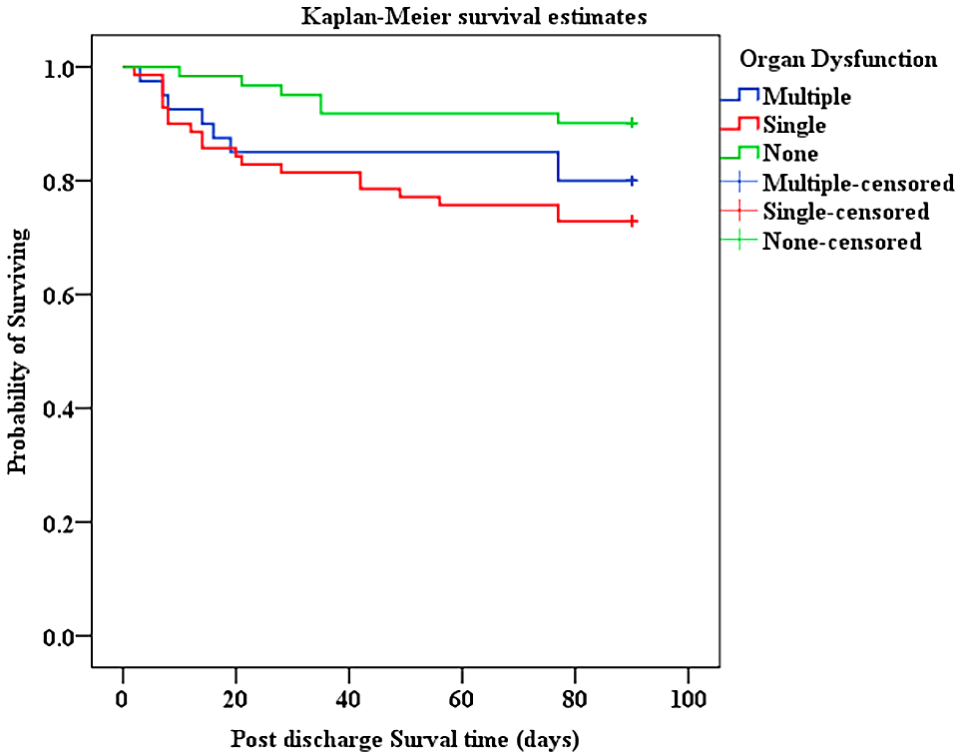
Kaplan-Meier survival curve for children with and without organ dysfunction who were discharged from PICU. **Log rank test**, *P* value < 0.040

**Fig. 5:**
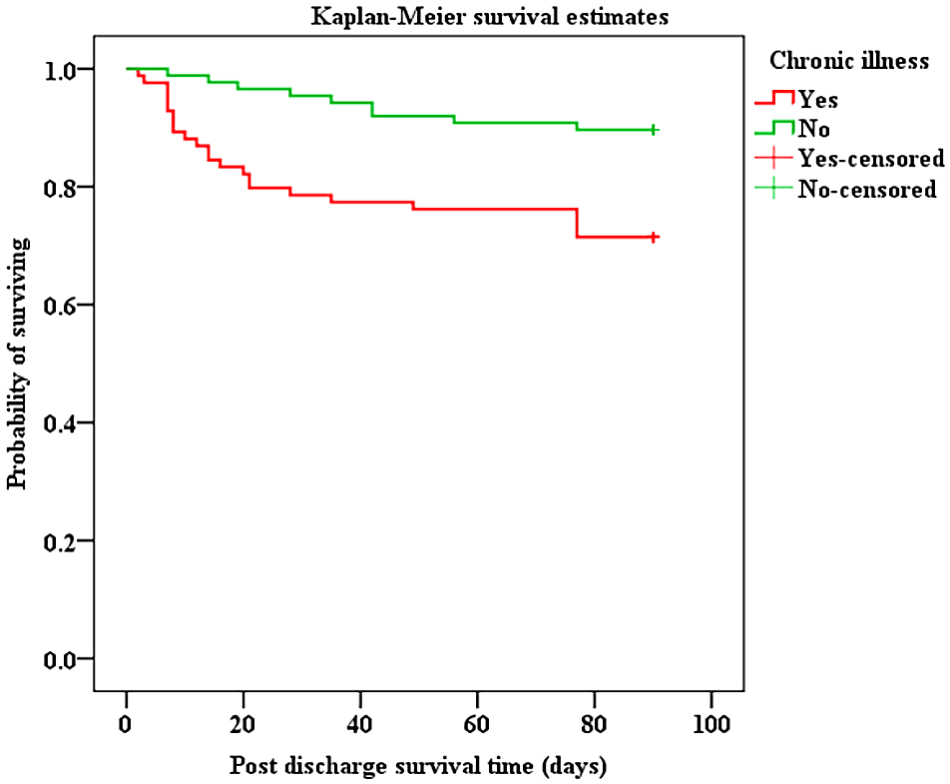
Survival curve for children with and without chronic illness who were discharged from PICU. **Log rank test**, *P* value < 0.020

Overall mortality from admission to three months after discharge was 49.8% (161/323) [MR = 10.74 per 1000-person days], 40%(128/323) of participants died in PICU and 10%(33/323) died within three months after discharge. The majority 79.5%(128/161) of deaths occurred in PICU, with a significant proportion 20.5%(33/161) occurring after discharge as shown in Fig. 2. Of 173 children discharged from PICU, 33(19.1%) died [MR = 2.5/1000–person days], with 51.5% (17/33) of deaths occurring in the general ward or as readmission to PICU but before hospital discharge. Majority of deaths 23/33 (70%) noted after PICU discharge occurred within 30 days and survival decreased to 80.7% at the end of follow-up period, Fig. 3. Children with organ dysfunction and chronic illness were more likely to die after PICU discharge with *p* = 0.040 and *p* = 0.002 respectively as indicated in Figs. 4 and 5.

.

### Causes of death after PICU discharge

**Fig. 6:**
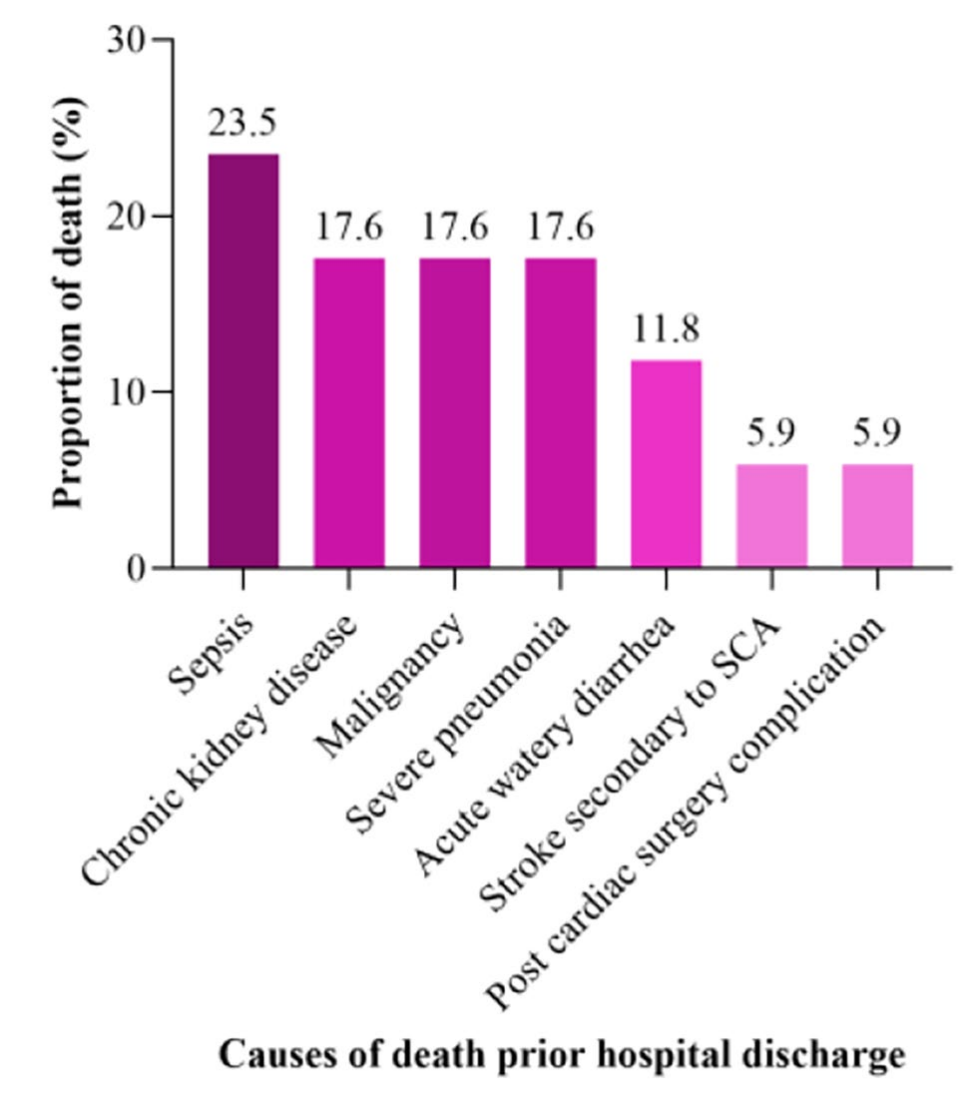
Cause of death prior to hospital discharge

Underlying causes of death among children who died before hospital discharged, either in the general ward or as a readmission to PICU included sepsis 4/17(23.5%), malignancy 3/17(17.6%), chronic kidney disease 3/17(17.6%), and severe pneumonia 3/17(17.6%) as shown in Fig. 6.

**Fig. 7:**
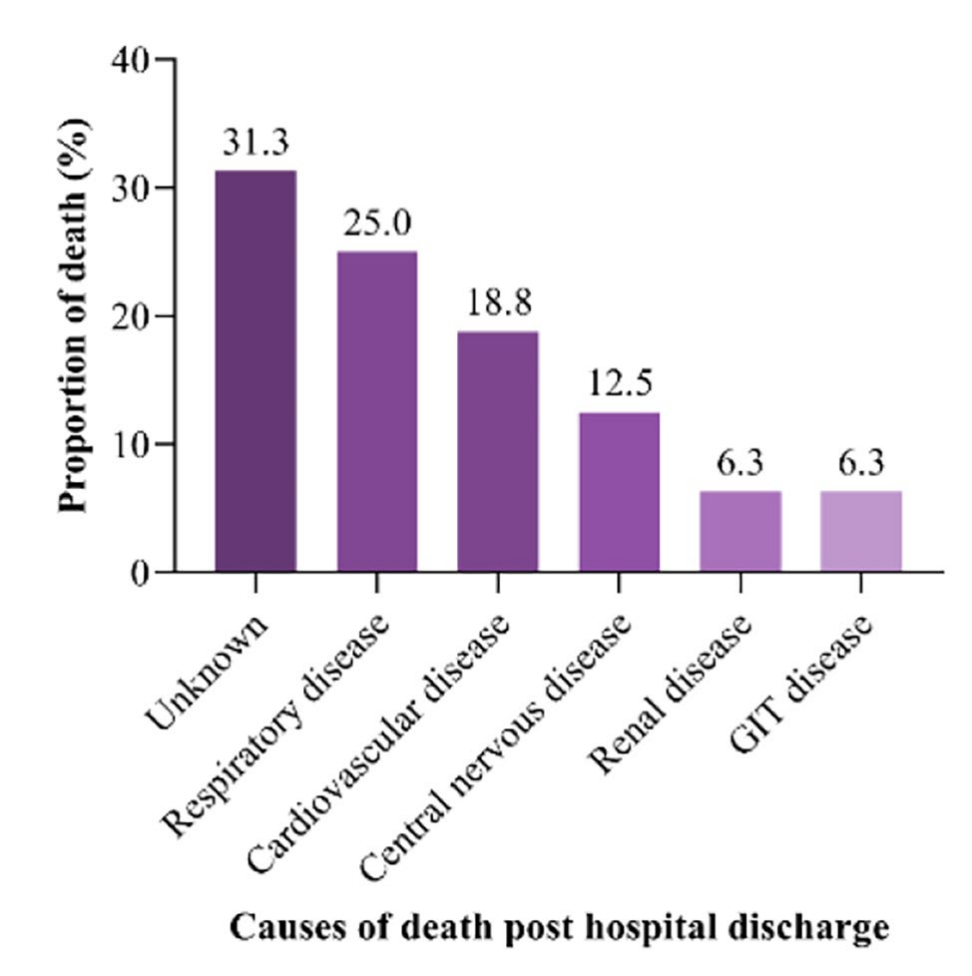
Cause of death after hospital Discharge

Causes of mortality after hospital discharge included diseases affecting respiratory 4/16(25.0%), cardiovascular 3/16(18.8%) and central nervous 2/16(12.5%) systems as shown in Fig. 7. Cause of death after hospital discharge could not be established in 5/16(31.3%) children.

### Factors associated with overall mortality during the study period

A higher hazard ratio for mortality was noted among participants with infectious disease [HR:1.65,95CI(1.18–2.30)], medical diagnosis [HR:2.07,95%CI(1.37–3.13)], multiple [(HR:5.71,95%CI(3.35–9.73)] and single [(HR:2.54,95%CI(1.44–4.49)] organ dysfunction. Significantly higher hazard ratio of dying was also noted among children with elevated serum creatinine [HR:1.87,95%CI(1.36–2.58)], elevated ALT [HR:1.71,95%CI(1.20–2.43)], and anemia [HR:1.70,95%CI(1.15–2.50)].

Admitting diagnosis, organ dysfunction, creatinine, ALT and hemoglobin were the variables included in multivariate analysis. After adjusting for confounders, multiple [HR:5.97,95%CI(3.22–12.06)] and single organ dysfunction [HR:2.77,95%CI(1.50–5.14)] were observed to independently predict overall mortality,, Table 2.

**Table 2:** Univariate and multivariate analysis of the factor associated with overall mortality of children admitted to PICU, from the time of admission to three months after discharge

		Univariate analysis			Multivariate analysis		
Variable		Crude HR	95% CI	*P*-value	Adjusted HR	95% CI	*P*-value
Age (Years)							
< 1		1.20	0.72–1.67	0.674			
1–5		0.95	0.63–1.45	0.820			
> 5		Ref					
Admiting diagnosis							
Infectious		1.65	1.18–2.30	0.004	0.76	0.52–1.12	0.163
Non Infectious		Ref					
Type of admission							
Medical		2.07	1.37–3.13	0.001			
Surgical		Ref					
Chronic Illness							
Yes		0.93	0.68–1.28	0.669			
No		Ref					
Organ Dysfunction							
Multiple		5.71	3.35–9.73	< 0.001	5.97	3.22– 12.06	< 0.001
Single		2.54	1.44–4.49	0.001	2.77	1.50–5.14	0.001
None		Ref					
Creatinine (micromol/L)							
Elevated		1.87	1.36–2.58	< 0.001	1.27	0.90–1.78	0.18
Normal		Ref					
ALT (units/L)							
>114		1.71	1.20–2.43	0.003	0.98	0.67–1.42	0.896
< 114		Ref					
Hemoglobin (g/dl)							
<11		1.70	1.15–2.50	0.008	1.28	0.85–1.91	0.238
>11		Ref					
Platelet (×10^9^/L)							
<150		1.21	0.86–1.70	0.271			
>450		0.69	0.41–1.16	0.156			
150–450		Ref					
WBC (×10^9^/L)							
<4		1.25	0.45–3.44	0.668			
>11		1.29	0.94–1.79	0.120			
4–11		Ref					

**ALT**: Alanine aminotransferase, **WBC**: White blood cells, **CI**: Confidence interval, **HR**: Hazard ratio

### Factors associated with mortality after PICU discharge

Factors associated with post-PICU discharge included presence of chronic illness [(HR:3.13,95%CI(1.46–6.74)], single organ dysfunction [HR:3.09,95%CI(1.23–7.74)] and thrombocytosis [HR:2.39,95%CI(1.03–5.51)].

Organ dysfunction, chronic illness and platelets were the variables included in multivariate analysis. After adjusting for confounders, independent predictors of mortality were chronic illness [HR:3.92,95%CI(1.77–8.66)], multiple organ dysfunction [HR:3.11,95%CI(1.01–9.61)], single organ dysfunction [HR:3.57,95%CI(1.42–9.03)] and thrombocytosis [HR:3.43,95%CI(1.46–8.05)], Table 3.

**Table 3:** Univariate and multivariate analysis of factors associated with mortality after PICU discharge

		Univariate analysis			Multivariate analysis		
Variable		Crude HR	95% CI	*P*-value	Adjusted HR	95% CI	*P*-value
Age (Years)							
< 1		0.74	0.30–1.77	0.493			
1–5		0.71	0.30–1.66	0.429			
> 5		Ref					
Admitting Diagnosis							
Infectious		1.07	0.54–2.13	0.837			
Non Infectious		Ref					
Type of admission							
Medical		2.31	0.95–5.59	0.064			
Surgical		Ref					
Chronic Illness							
Yes		3.13	1.46–6.74	0.004	3.92	1.77–8.66	0.001
No		Ref					
Organ Dysfunction							
Multiple		2.19	0.76–6.31	0.147	3.11	1.01–9.61	0.049
Single		3.09	1.23–7.74	0.016	3.57	1.42 - 9.03	0.007
None		Ref					
Creatinine (micromol/L)							
Elevated		1.32	0.66–2.65	0.432			
Normal		Ref					
ALT (units/L)							
>114		1.60	0.66–3.87	0.299			
< 114		Ref					
Hemoglobin (g/dl)							
< 11		0.88	0.43–1.78	0.712			
>11		Ref					
Platelets (×10^9^/L)							
< 150		1.80	0.80–4.04	0.158	1.74	0.74–4.05	0.203
>450		2.39	1.03–5.51	0.042	3.43	1.46–8.05	0.005
150–450		Ref					
WBC (×10^9^/L)							
< 4		3.26	0.42–25.47	0.260			
>11		1.92	0.91–4.06	0.090			
4–11		Ref					

**ALT**: Alanine aminotransferase, **WBC**: White blood cells, **CI**: Confidence interval, **HR**: Hazard ratio

## Discussion

### Mortality of children after admission to pediatric intensive care unit

This study sought to ascertain the mortality rates of children admitted to PICU from the point of admission up to three months’ post- discharge, along with the factors influencing this outcome. The overall mortality among these children was 49.8% and mortality after discharge from PICU was 19.1%. Independent predictors of mortality encompassed the presence of organ dysfunction, chronic illness, and thrombocytosis.

The study revealed a high overall mortality of 49.8%, surpassing the findings of a prior study conducted at Hippokratio General Hospital in Thessaloniki, Greece with a mortality of 15% at three month of following up 300 children aged between 6 weeks to 14 years admitted to PICU [4]. The observed disparity could potentially be attributed to variations in economic status between the two study areas, influencing the availability and utilization of medical technology and, consequently, impacting patient outcomes.

In-PICU mortality in our study was 40%, exceeding the mortality observed in studies conducted in African countries such as Mozambique, Ethiopia, and South Africa, where mortality of 25%, 8.5%, and 15.6%, respectively, were documented [2, 14, 15]. This discrepancy may be due to the retrospective nature of the prior studies conducted in African settings, involving children aged 1 month to 14 years admitted to the PICU for either medical or surgical reasons [2, 14, 15].

Furthermore, a notable 10% of the participant succumbed within three month post- PICU discharge, signifying a higher mortality than that observed at Hippokratio General Hospital in Thessaloniki, Greece, where a mortality of 5.3% was documented during the same three-month follow-up period [4]. This difference might be elucidated by Greece’s status as a developed country, benefiting from advanced medical technology and thereby yielding improved clinical outcomes.

The present study revealed a post-PICU discharge mortality rate of 19.1%, markedly surpassing the 1.9% mortality reported in United Kingdom (UK), where a cohort of 2,165 children admitted to pediatric intensive care units in Wales, England, and Scotland were followed up for a 6-month period [5]. These disparities may be ascribed to variations in the age criteria employed, with the UK study excluding children below 6 months of age, while our study included this age group.

Furthermore, in the aforementioned UK study, a comparative analysis between participants who provided consent and those who did not revealed that the non-consenting group exhibited greater illness severity. While our study did not conduct a similar comparison, we noted a high level of illness severity in our participants, as indicated by the prevalence of organ dysfunction. These distinctions could account for the higher mortality in our study. Additionally, UK being a developed country with advanced medical technology, may contribute to better patient outcomes.

Similar to study done in Thessaloniki, Greece [4], our our research revealed a significant pattern of post-PICU discharge mortality, with a slightly higher occurrence before hospital discharge. In our study, 10.6% of deaths transpired prior to hospital discharge, compared to 9.9% post-hospital discharge. In Greece, 3% occurred before hospital discharge and 2.3% after hospital discharge. The observed pattern may be linked to the absence of high dependence unit (HDU) in both our setting and Greece, which is crucial in stabilization of patient discharged from PICU.

Three month-survival rate among participants in our study following PICU discharge was 80.7%, surpassing the 3-year survival rate of 75% observed by Matsumoto et al. in a study conducted in Osaka, Japan, involving 102 children aged < 15 years admitted in PICU with prolonged stay [6]. This indicates a continued decline in survival for patients discharged from the PICU, extending beyond the initial three-month follow-up period.

### Factors associated with mortality

In our study, the presence of organ dysfunction independently predicted both overall mortality and post-PICU discharge mortality. A comparable influence of organ dysfunction was noted in previous study in the United States (U.S), which obtained data on children admitted to various PICUs from a database [16]. This can be explained by the idea that the occurrence of organ dysfunction indicates the severity and advance stage of an illness, resulting in either immediate mortality or presence of sequelae that increase the long-term risk of mortality.

Similar to the previous investigation conducted by Hau et al. in Tanzania, focusing on post-hospital mortality, our study identified chronic illness as as independent predictor of mortality following PICU discharge [9]. This highlights the vulnerability of children with chronic illness and emphasizes the necessity for the development of specialized care and intervention plans to enhance their overall outcome.

In our study, we noted thrombocytosis as independent predictor of mortality after PICU discharge. There is a scarcity of published data on the impact of thrombocytosis specifically in critically ill children or those who have recovered from critical illness. However, a study conducted by Ghoneim et al. at Zagazig University Hospital, Egypt [17], focusing on hospitalized adult patients with community-acquired pneumonia, identified thrombocytosis as one of the predictors of mortality within first 30 days after admission.

The precise mechanism underlying the association between thrombocytosis and mortality remains not fully elucidated. Nevertheless, a study conducted by Ghoneim et al. [17].

demostrated life-threatening complications, such as pleural effusion, in adult patients with thrombocytosis admitted due to community-acquired pneumonia.

Furthermore, a study done by Sreenivasa et al. in Bangalore, involving 178 children aged 2 months to 5 year admitted in hospital due to lower respiratory tract infection, revealed an elevation in thrombocytosis with increasing pneumonia severity [18]. Even though the direct link of thrombocytosis to mortality has not been established, the observations from the aforementioned studies may imply that thrombocytosis reflects an inflamation which lead to a severe and complicated acute respiratory illness with potentially long-term sequelae impacting survival.

### Causes of death after PICU discharge

Underlining cause of death was found in the medical records for the participants who died before hospital discharge in our study. Sepsis, malignancy, chronic kidney disease, and severe pneumonia were the leading cause of death. This was different from the study done in Thesaloniki,Greece among 300 children admitted to PICU where the leading cause of death was post-operative care [4]. This could be because of high burden of infectious disease and antimicrobial resistance in Sub-Saharan Africa than Greece [19, 20].

Cause of death after hospital discharge was determined by physician-certified verbal autopsy. Respiratory, cardiovascular, and central nervous system diseases were the leading causes of death after hospital discharge. Similar findings were observed in previous study done in Thesaloniki, Greece were cause of death at three month after PICU discharge was respiratory, cardiac, and central nervous system related [4]. In our study, the cause of death remained undetermined in 31% of participants when utilizing verbal autopsy. A comparable difficulty was noted in investigations conducted in Mexico, Indonesia, and Rwanda, where the cause of death could not be determined in 2%, 17.2%, and 9.5% of children, respectively, through verbal autopsy [21–23, 4].

### Limitation

The use of one physician in verbal autopsy subjected the findings to potential bias. Emotional distress and over the phone administration of verbal autopsy hindered acquisition of information on the cause of death for 31% of participants. Over the phone interview made it difficult to make a conversation longer enough to narrow down the cause of deaths.

Our Modified verbal autopsy captured the event around death and this could lead into missing important information required to determine immediate cause of death. It was difficult to be certain if the cause of death was related to PICU admission in most cases. Additionally, some of the factors such as malnutrition and PICU readmission which influence the outcome of critically ill children were not well studied.

### Conclusion and recommendations

The overall mortality in our study was high and significant proportion of participant die in PICU as well as after discharge. Approximately half of the mortality after PICU discharge occurred before hospital discharge and majority of them occurred within first 30days. Organ dysfunction, chronic illness and thrombocytosis independently predicted mortality.

We recommend an establishment of HDU to keep patients discharged from PICU under close observation before transfer them to general ward. Focused and individualized follow up plan of these patients is needed after discharge. We also recommend further research on long term survival of patients discharged from PICU, factors present after discharge which may affect their survival, exactly cause of death and its relation to PICU admission. Lastly, we recommend research on intervention measures which could improve outcome of these patients.

## Data Availability

The data set generated and analyzed in this study are available from corresponding author on a reasonable request.

## Abbreviations

ALT: Alanine Amino Transference
GIT: Gastrointestinal Tract
Hb: Haemoglobin
HR: Hazard Ratio
ICU: Intensive Care Unit
MUHAS: Muhimbili University of Health and Allied Sciences
MNH: Muhimbili National Hospital
PICU: Pediatric Intensive Care Unit
WBC: White Blood Cell
WHO: World Health Organization

## References

[1] Ibiebele I, Algert CS, Bowen JR, Roberts CL. Pediatric admissions that include intensive care: a population-based study. 2018;1–8. 10.1186/s12913-018-3041-x. 10.1186/s12913-018-3041-x PMC5892018 29631570

[2] Punchak M, Hall K, Seni A, Buck WC, Deugarte DA, Hartford E (2018). Epidemiology of diseases and mortality from a PICU in Mozambique. Pediatr Crit Care Med.

[3] Abdelatif RG, Mohammed MM, Mahmoud RA, Bakheet MAM, Gima M, Nakagawa S (2020). Characterization and outcome of two Pediatric Intensive Care units with different resources. Crit Care Res Pract.

[4] Volakli EA, Sdougka M, Drossou-Agakidou V, Emporiadou M, Reizoglou M, Giala M (2012). Short-term and long-term mortality following pediatric intensive care. Pediatr Int.

[5] Jones S, Rantell K, Stevens K, Colwell B, Ratcliffe JR, Holland P (2006). Outcome at 6 months after admission for pediatric intensive care: a report of a national study of pediatric intensive care units in the United Kingdom. Pediatrics.

[6] Matsumoto N, Hatachi T, Inata Y, Shimizu Y, Takeuchi M (2019). Long-term mortality and functional outcome after prolonged paediatric intensive care unit stay. Eur J Pediatr.

[7] Wiens MO, Bone JN, Kumbakumba E, Businge S, Tagoola A (2023). Post-discharge mortality among children under 5 years admitted with suspected sepsis in Uganda: a prospective multi-site, observational cohort study. Lancet Child Adolesc Health.

[8] Moisi JC, Gatakaa H, Berkley JA, Maitland K, Mturi N, Newton CR (2011). Excess child mortality after discharge from hospital in Kilifi, Kenya: a retrospective cohort analysis. Bull World Health Organ.

[9] Hau DK, Chami N, Duncan A, Smart LR, Hokororo A, Kayange NM (2018). Post-hospital mortality in children aged 2–12 years in Tanzania: a prospective cohort study. PLoS ONE.

[10] Nemetchek B, English L, Kissoon N, Ansermino JM, Moschovis PP, Kabakyenga J, et al. Paediatric postdischarge mortality in developing countries: a systematic review. BMJ Open. 2018;8(12). 10.1136/bmjopen-2018-023445. 10.1136/bmjopen-2018-023445 PMC6318528 30593550

[11] Goldstein B, Giroir B, Randolph A. International pediatric sepsis consensus conference: definitions for sepsis and organ dysfunction in pediatrics. Pediatr Crit Care Med. 2005;6(1). 10.1097/01.PCC.0000149131.72248.E6. 10.1097/01.PCC.0000149131.72248.E6 15636651

[12] Proulx F, Fayon M, Farrell CA, Lacroix J, Gauthier M (1996). Epidemiology of sepsis and multiple organ dysfunction syndrome in children. Chest.

[13] 2022 WHO Verbal Autopsy Instrument.[Internet]. 2022[cited 2022 April 24]. Available fromhttps://www.who.int/publications/m/item/2022-who-verbal-autopsy-instrumet.

[14] Haftu H, Hailu T, Medhaniye A, Teklit G (2018). Assessment of pattern and treatment outcome of patients admitted to pediatric intensive care unit, Ayder Referral Hospital,Tigray, Ethiopia. BMC Res Notes.

[15] Hendricks CL, Mckerrow NH, Hendricks RJ (2016). Factors present on admission associated with increased mortality in children admitted to a paediatric intensive care unit (PICU). South Afr J Child Health.

[16] Typpo KV, Petersen NJ, Hallman DM, Markovitz BP, Mariscalco MM (2009). Day one MODS is associated with poor functional outcome and mortality in Pediatric Intensive Care Unit. Pediatr Crit Care Med.

[17] Ghoneim AHA, Mohammad MA, Elghamrawy MA, Embarak S (2020). Platelet count as a predictor of outcome of hospitalized patients with community-acquired pneumonia at Zagazig University Hospitals, Egypt. Egypt J Bronchol.

[18] Sreenivasa B, Kumar GV, Manjunatha B (2015). Study of significance of thrombocytosis in lower respiratory tract infections in children. Int J Contemp Pediatr.

[19] Michaud CM. Global burden of infectious diseases. In: Moselio Schaechter, editor. Encyclopedia of Microbiology. Publication online: 2009. p 444–454. 10.1016/B978-012373944-5.00185-1.

[20] Murray CJ, Ikuta KS, Sharara F, Swetschinski L, Robles Aguilar G, Gray A (2022). Global burden of bacterial antimicrobial resistance in 2019: a systematic analysis. Lancet.

[21] Gupta N, Hirschhorn LR, Rwabukwisi FC, Drobac P, Sayizoga F, Mugeni C (2018). Causes of death and predictors of childhood mortality in Rwanda: a matched case-control study using verbal social autopsy. BMC Public Health.

[22] Ramirez-Villalobos D, Stewart AL, Romero M, Gomez S, Flaxman AD, Hernandez B (2019). Analysis of causes of death using verbal autopsies and vital registration in Hidalgo, Mexico. PLoS ONE.

[23] Wahab A, Choiriyyah I, Wilopo SA (2017). Determining the cause of death: mortality surveillance using verbal autopsy in Indonesia. Am J Trop Med Hyg.

