# Correction: Mortality and associated factors among children admitted to an intensive care unit in muhimbili national hospital, from the time of admission to three months after discharge: a prospective cohort study

**DOI:** 10.1186/s12887-024-04708-z

**Published:** 2024-03-20

**Authors:** Rehema E. Lyimo, Yasser H. Said, Sokoine L. Kivuyo, Deogratias Nkya, Francis F. Furia

**Affiliations:** 1 Department of Pediatrics and Child Health, School of Medicine, Muhimbili University of Health and Allied Sciences, Dar Es Salaam, Tanzania; 2 Department of Paediatrics and Child Health, Muhimbili National Hospital, Dar Es Salaam, Tanzania; 3 National Institute for Medical Research, Dar Es Salaam, Tanzania; 4 Department of Pediatric Cardiology, Jakaya Kikwete Cardiac Institute, Dar Es Salaam, Tanzania

**Correction: BMC Pediatr 24, 170 (2024)**

**https://doi.org/10.1186/s12887-024-04620-6**

Following the publication of the original article [1], the authors identified an error in Fig. 1. The correct and incorrect figures are given below.

The original article has been corrected.

Incorrect Fig. 1:
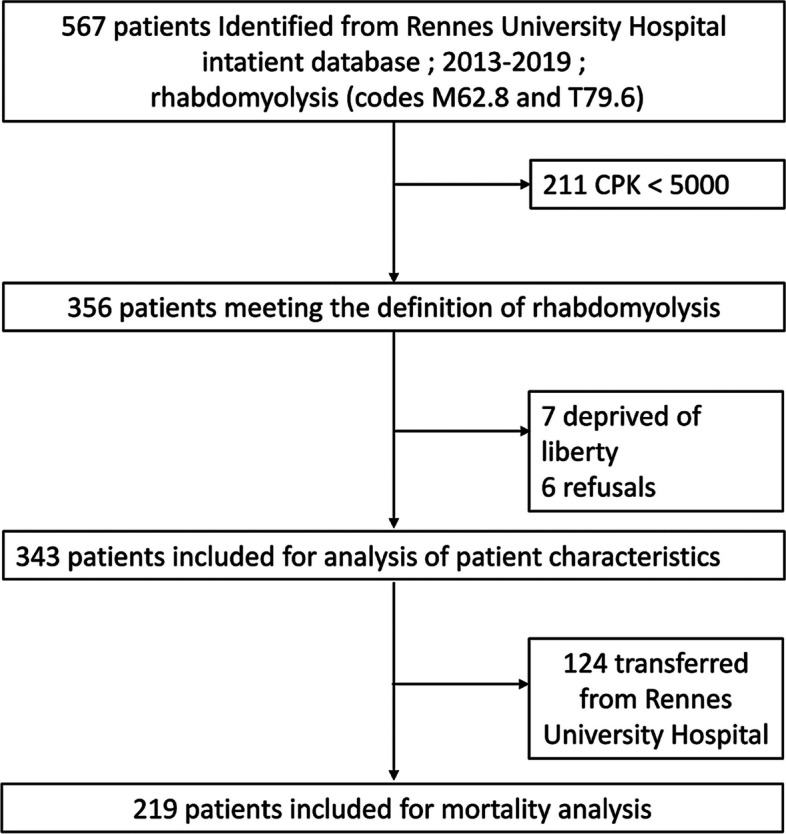


Correct Fig. 1:

**Fig. 1:**
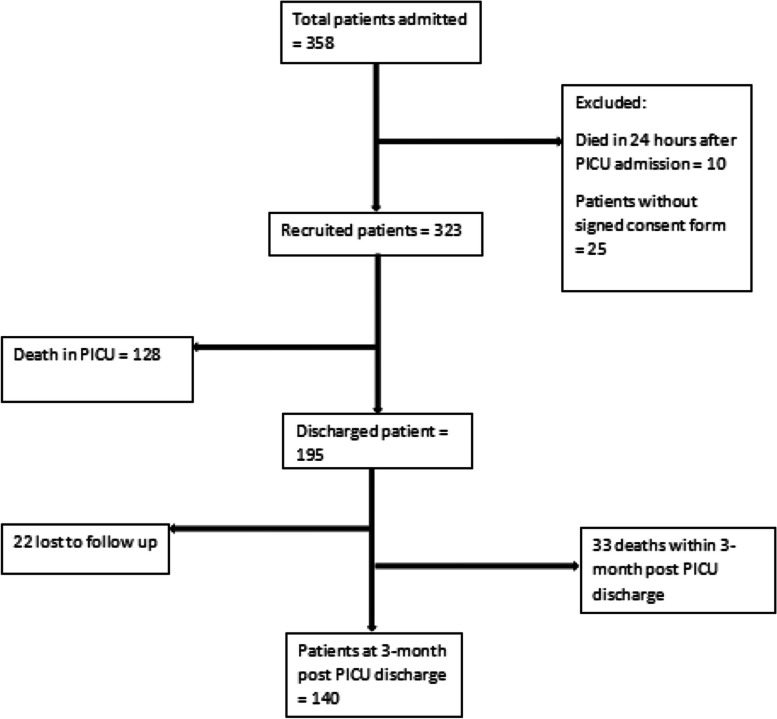
Flow chart on enrollment of study participants
